# Unveiling the veil: exploring how wellbeing motivations shape anonymous and public prosocial behavior in Indonesia

**DOI:** 10.1186/s40359-024-01799-2

**Published:** 2024-05-27

**Authors:** Livia Yuliawati

**Affiliations:** School of Psychology, Universitas Ciputra Surabaya, Citraland CBD Boulevard, Surabaya, 60219 Indonesia

**Keywords:** Hedonic, Eudaimonic, Extrinsic, Motives, Wellbeing, Prosocial behavior

## Abstract

Indonesia is often regarded as a country with a strong inclination toward prosocial behavior, with both public and anonymous acts of kindness being commonplace. However, there is a notable gap in related research regarding the predictors of such behaviors. Previous studies have highlighted how individuals with diverse motives for wellbeing are inclined to either assist or hinder others. The present study explored the role of eudaimonic, hedonic, and extrinsic motives for wellbeing in predicting public and anonymous prosocial behavior. Using convenience sampling, 254 Indonesian undergraduate students (18-25 years old) from a private university participated in an online survey. The data were analyzed with correlational design and structural equation modelling. The findings revealed that the eudaimonic motive for wellbeing positively predicted anonymous prosocial behavior. Interestingly, no significant impact of hedonic motives on either public or anonymous prosocial behavior was observed. On the other hand, the extrinsic motive for wellbeing emerged as a positive predictor of public prosocial behavior. The absence of a discernible effect of the hedonic motive on either form of prosocial behavior highlights the need for further research into the complex interplay between motives for well-being and altruistic actions. This research represents a pioneering exploration into the distinct impacts of individuals' pursuit of wellbeing on their approaches to altruistic actions, providing valuable insights for understanding and promoting prosocial behavior in society.

## Introduction

According to the World Giving Index, Indonesia has been ranked as the most generous country in the world for six consecutive years in terms of aid to foreigners, cash donations and volunteering [1]. According to a 2022 Expat Insider survey, Indonesia is the second most friendly country in the world where expatriates or foreign workers feel treated kindly as foreigners and do not find it difficult to make friends [2]. Anonymous donations during the COVID-19 outbreak were more widespread in Indonesia than in the U.S. [3]. Other data also showed that the participation of Indonesian Gen-Z (born between 1997 and 2012) in online donations increased [4]. During the pandemic, 24.78% of respondents volunteered more than 90 hours in total for various activities such as promoting health awareness campaigns, distributing health safety equipment, and initiating fundraising activities for marginalized communities [5]. Those data show that prosocial behavior is part of daily life in Indonesia, one of the most populous and religious countries in the world.

Prosocial behavior denotes a deliberate and purposeful action that contributes to the well-being of another individual [6, 7]. Consistently, prosocial behavior has been linked to a range of positive psychological outcomes. Drawing from Gallup World Poll data spanning 130 countries [8], it displayed that there was a correlation between prosocial spending and life satisfaction. Additionally, a recent meta-analysis indicated that engaging in prosocial behavior enhances overall well-being [8]. A longitudinal study spanning nine years found that individuals who consistently exhibited high levels of prosocial behavior from middle childhood to late adolescence demonstrated lower instances of externalizing behaviors, including aggression, cheating, and rule-breaking [9]. Remarkably, even toddlers experience heightened happiness through giving [10]. In the Indonesian context, possessing good character is perceived to affect one’s wellbeing [11]. Prosocial spending was found to have a positive impact on subjective well-being [12]. Moreover, participation in prosocial behavior has been linked to enhanced psychological well-being among Indonesian adolescents [13].

Several research have demonstrated that the motives driving prosocial behavior significantly influence its outcomes and the nature of helping behavior. For instance, individuals who engage in helping others with intrinsic motivation tend to experience greater well-being than do those who feel obligated to help [14]. Recalling experiences of helping with an other-focused motive elicits more positive emotions than when the motivation is self-focused [15]. A separate longitudinal study showed that volunteers motivated by an other-oriented mindset tended to outlive nonvolunteers, while those motivated by a desire for personal benefit did not enjoy the same longevity advantage [16]. Distinct personal values have been identified as predictors of varying types of donations, whether for environmental causes, religious communities, or animal welfare [17].

Individuals may harbor diverse motives in their quest for well-being, as outlined by a prior study [18]. These motives can be categorized into hedonic, eudaimonic, and extrinsic dimensions. Those guided by hedonic motives typically pursue pleasure and comfort. Conversely, eudaimonic motives encompass a commitment to self-discovery and authenticity, adherence to moral standards and excellence, and a drive for personal growth and the cultivation of purpose and significance. On the other end of the spectrum lies extrinsic motivation, where individuals pursue material possessions, power, fame, and prestige. Several studies have delved into the correlation between motives in the pursuit of well-being and coping strategies. Notably, another research showed a positive correlation between eudaimonic motives and adaptive coping strategies, while hedonic motives were associated with relaxation as an avoidant coping strategy [19]. Notably, only eudaimonic motives emerged as a predictor of both subjective and psychological well-being [20]. Furthermore, eudaimonic motives have been identified as positive predictors of academic achievement and negative predictors of negative emotions, contrasting with hedonic motives, which did not contribute significantly to these outcomes [21]. Despite the extensive exploration of these motives, the specific role of hedonic, eudaimonic, and extrinsic motives in shaping prosocial behavior in both public and anonymous settings remains unclear in existing research.

The following research on prosocial behavior within diverse samples in Indonesia have illuminated various predictors. Notably, empathy emerges as a robust positive predictor of prosocial behavior and is evident among both nurses [22] and elementary school students [23]. The intricate landscape of prosocial behavior among Indonesian adolescents unveils a multifaceted interplay of factors, including emotional maturity [24], emotional intelligence, and religiosity [25, 26], in addition to adhering to collectivistic values [27]. Furthermore, gratitude has been identified as significantly associated with prosocial behavior among undergraduate students [28]. Expanding the scope nationally, a comprehensive study encompassing samples from all provinces in Indonesia delves into the predictors of prosocial behavior. This investigation highlights the significant predictive role of happiness, meaning in life, trust, tolerance, age, and income as significant predictors [29].

Indonesia has been ranked as the 12^th^ country in happiness index, in which there are 79% of Indonesians perceiving that they are happy [30]. Unfortunately, the literature has paid limited attention to how motives in the pursuit of well-being impact public and anonymous prosocial behavior in Indonesia. Thus, the present study aims to rectify this research gap by exploring the nuanced dynamics of eudaimonic, hedonic, and extrinsic motives in the pursuit of well-being and their influence on public and anonymous prosocial behavior in Indonesia.

Despite Indonesia's high ranking in the happiness index, there is limited research on this topic in the context of Indonesia specifically. By focusing on eudaimonic, hedonic, and extrinsic motives, the present study is expected to provide a nuanced understanding of how these motives shape individuals' behaviors towards others in both public and anonymous settings. This research is important as it can shed light on the factors that contribute to the high levels of happiness and prosocial behavior reported in Indonesia as well as provide insights for further research on how to promote prosocial behavior.

### Eudaimonic, hedonic, and extrinsic motives of happiness and prosocial behavior

Different motives of happiness play different impact on prosocial behavior. Individuals adopting a eudaimonic approach derive greater enjoyment from engaging in prosocial behavior than do those with a hedonic orientation [31]. A study among Chinese adolescent revealed that pleasure orientation and meaning orientation were positively associated with prosocial behavior [32]. Individuals possessing self-transcendence values (benevolence, universalism), which are close to eudamonic values, were more likely to have a prosocial orientation while holding hedonic and extrinsic motives, represented in self-enhancement values, such as achievement and power had no significant correlation with prosocial orientation [33]. Hedonic orientation was not substantially correlated with helping or hindering others but eudaimonic orientation was positively connected to assisting others and adversely related to impeding others [34].

Moreover, the motivations driving the pursuit of well-being can influence the choice between publicly or anonymously displaying prosocial behavior. Prosocial actions can unfold either in a public setting or in an anonymous environment. Public prosocial behavior involves assisting others with the aim of gaining approval from others [35]. There are some individuals who prefer providing their help without being identified by others or by beneficiaries, that is called anonymous prosocial behavior. Another research discovered that individuals with hedonistic motives only extend a helping hand in specific situations—when they can visually perceive the recipient, when the outcomes are apparent, and when there is no personal cost involved [34] In contrast, those with eudaimonic inclinations demonstrate readiness to assist others even in scenarios where the benefits may not be immediate, the recipient remains unseen, or personal sacrifice is needed.

A research in Indonesia uncovered a compelling phenomenon wherein self-identified donors contributed significantly more money on online donation platforms than did their anonymous counterparts [3]. An experimental study demonstrated that individuals with a high interdependent self-construal were inclined to make donations when their actions could be observed in a public context, driven by a desire to portray themselves as caring individuals [36]. Conversely, a recent study revealed that individuals with a collectivistic orientation place greater emphasis on the benefits to others, while individuals with an individualistic orientation engage in prosocial behavior primarily for personal gain [37]. Hedonism exhibited a positive correlation with prosocial activities, particularly when the recipient was depicted as a significant other. In contrast, eudaimonia was associated with greater donations to broader or more far-reaching causes [38].

Engaging in prosocial behavior enhances the perception of individuals possessing favorable qualities [39]. Consequently, carrying out prosocial acts in public is a strategic move to elevate one's social reputation [40] and to showcase positive qualities to attract potential cooperative partners or allies, as outlined by reputation-based partner choice theory [41]. Despite these potential benefits, there is a risk that assisting others may come with social costs, such as exclusion or defamation [42]. Consequently, individuals may opt to conceal their prosocial deeds to safeguard their reputation. An underlying reason for engaging in anonymous prosocial behavior could be the desire to avoid the discomfort associated with public exposure, as this may lead to a diminished sense of moral virtue and reputation in the eyes of others [43].

Receiving recognition has been shown to boost monetary donations, especially among individuals with high extrinsic religiosity [44]. The motivation to expect rewards appears to influence how individuals engage in prosocial behavior. According to Schwartz's values, individuals with self-transcendence values are more likely to exhibit prosocial orientation, while this tendency is not observed among those with self-enhancement values such as power and fame [33]. However, some research suggests that pursuing extrinsic motives such as power and material wealth may indeed predict prosocial behavior. In situations where individuals with a high power motive know that their actions are visible in a public context, they are more inclined to engage in prosocial behavior [45]. A study involving adults showed that materialism influenced the impact of accounting for time on prosocial behavior [46]. Specifically, accounting for time reduced the time and money spent on others for participants with moderate levels of materialism, but not for those with low or high levels of materialism [47]. It is argued that materialism can lead to prosocial behavior, potentially enhancing one's status. Conversely, materialism can hinder prosocial behavior due to its inherent focus on self-interest rather than the needs of others [46].

Drawing from these explanations, the eudaimonic motive tends to align with self-transcendence values, reflecting a willingness to help even those who are not deemed significant others. Individuals with this motive might opt to conceal their prosocial actions, fearing potential harm to their moral identity. Consequently, it is hypothesized that eudaimonic motives will exert a more pronounced influence on prosocial behavior in anonymous contexts than in public settings (Hypothesis 1). In contrast, individuals driven by hedonic and extrinsic motives are more likely to engage in prosocial actions when directed toward significant others, especially if such actions promise personal benefits and rewards. Therefore, it is proposed that hedonic motives for happiness (Hypothesis 2) and extrinsic motives (Hypothesis 3) will have stronger impact on prosocial behavior in public contexts than in anonymous settings.

## Method

### Participants

Indonesia has a diverse range of universities, including public, private, and religious institutions. From around 4002 universities in Indonesia, 95.4% of them are private universities [48]. Employing convenience sampling by sending the invitation to participate in the research, there were 254 undergraduate students who joined as participants from a private university in Indonesia with total population was approximately 5000 students. They were 40.2% from business major, 16.2% from tourism major, 15.6% from design major, and the rest of them were majoring in psychology, communication, informatics, and medicine. The participants mainly came from the second (20.1%), the third (26.3%) and fourth years of study (33%).

The participants received an online survey and were provided a lucky draw, offering an e-wallet equivalent to USD 3. All 254 undergraduate students consented to participate in the research and were assured of the anonymity and voluntary nature of their participation. Participants retained the option to withdraw from the activity at any point without facing any consequences. Notably, 68.2% of the participants were female and aged 18 to 25 years.

### Measures

#### Hedonic, Eudaimonic, and Extrinsic Motives for Activities (HEEMA)

This 16-item scale measures individual’s motivation to pursue wellbeing and can be categorized into hedonic, eudaimonic, and extrinsic dimensions [49]. The hedonic dimension comprises six items measuring pleasure and comfort motives. The eudaimonic dimension with five items assesses motives related to personal growth and meaning. Extrinsic motive is examined with five items focusing on power, popularity, and wealth. All those dimensions had good internal consistency with Cronbach’s α ranging from 0.79 to 0.91 [49]. Each participant was asked the following question “To what extent do you typically approach your activities with each following intentions, whether or not you actually achieve your aim?” Participants rated each item on a 7-point Likert scale (1=not at all; 7= very much). In the current study, the eudaimonic motive (Cronbach’s alpha=.84), hedonic motive (Cronbach’s alpha=.84), and extrinsic motive (Cronbach’s alpha=.85) items in the present study have good reliability.

#### Prosocial tendencies measure-public and anonymous dimensions

The original version of Prosocial Tendencies Measure has good internal consistency [35]. The public dimension contains four items with a Cronbach’s α = 0.78 while the anonymous dimension with 5 items had a Cronbach’s α = 0.85 On a 5-point Likert scale from 1 (does not describe me at all) to 5 (describes me substantially), participants were asked to rate how much the statements described them. This scale has been use to different countries, including Indonesia [50–52]. In the present study, Cronbach’s alpha for public prosocial behavior was .74 while anonymous prosocial behavior also had good internal consistency with Cronbach’s alpha=.68.

## Results and discussion

Data were cleaned and coded before analyses. Data analyses were performed using JASP 0.18.1.0. Confirmatory factor analyses were performed for each scale. Both Hedonic, Eudaimonic, and Extrinsic Motives for Activities scale (R-CFI=.95, R-TLI=.935, R-RMSEA=.07, SRMR=.06) as well as Prosocial Tendencies Measure-Public and Anonymous Dimensions had an acceptable fit to the data (R-CFI=.94, R-TLI=.92, R-RMSEA=.06, SRMR=.06) [53]. The data were not normally distributed (Mardia’s skewness coefficient=747.903, Mardia’s kurtosis coefficient=2358.05, *p*<.001). Robust error calculations were used. Correlations between variables were analyzed.

**Fig. 1:**
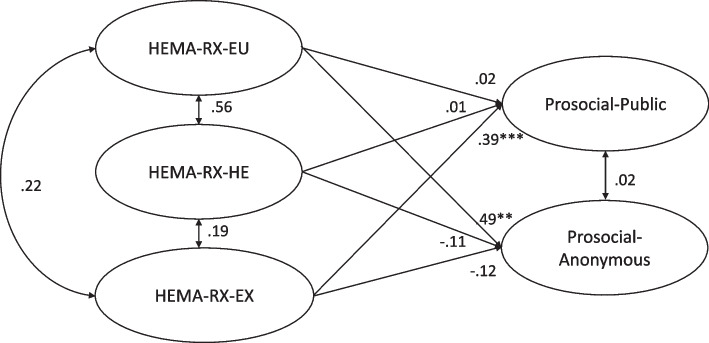
The role of eudaimonic, hedonic, extrinsic motives on public and anonymous prosocial behavior. Note: *p<.05 **p<.01 ***p<.001 HEEMA-EU= Hedonic, Eudaimonic, and Extrinsic Motives for Activities-Eudaimonic Motive; HEEMA-HE= Hedonic, Eudaimonic, and Extrinsic Motives for Activities-Hedonic Motive; HEEMA-EX= Hedonic, Eudaimonic, and Extrinsic Motives for Activities-Extrinsic Motive. All standardized regression coefficients are presented

As outlined in Table 1, there were notable positive correlations between eudaimonic and hedonic motives (*ρ*=.52, *p<.*001), hedonic and extrinsic motives (*ρ*=.33, *p<.*001), as well as eudaimonic and extrinsic motives (*ρ*=.28, *p<.*001). Particularly noteworthy is the stronger positive correlation observed between the eudaimonic motive and anonymous prosocial behavior (*ρ*=.32, *p<.*001) than between the correlation between the eudaimonic motive and public prosocial behavior (*ρ*=.17, *p<.*05). Interestingly, neither public (*ρ*=.32, *p=*.43) nor anonymous prosocial behavior (*ρ*=.32, *p=*.59) exhibited a correlation with hedonic motive. The extrinsic motive, on the other hand, demonstrated a positive link exclusively with public prosocial behavior (*ρ*=.33, *p<.*001) Fig. 1.

**Table 1:** Descriptive statistics and correlations of all variables

**Variables**	**1**	**2**	**3**	**4**	**5**
1. HEEMA-EU					
2. HEEMA-HE	.52***				
3. HEEMA-EX	.28***	.33***			
4. PROSOCIAL-P	.17*	.07	.33***		
5. PROSOCIAL-A	.32***	.11	.03	.15	
*M*	29.68	28.77	31.03	12.25	19.32
*SD*	4.42	4.51	6.41	3.63	3.17

*HEEMA-EU* Hedonic, Eudaimonic, and Extrinsic Motives for Activities-Eudaimonic Motive, *HEEMA-HE* Hedonic, Eudaimonic, and Extrinsic Motives for Activities-Hedonic Motive, *HEEMA-EX* Hedonic, Eudaimonic, and Extrinsic Motives for Activities-Extrinsic Motive/ *PROSOCIAL-P* Public Prosocial Behavior, *PROSOCIAL-A* Anonymous Prosocial Behavior

^*^p<.05 ***p<.001 All correlation coefficients are Spearman’s rho coefficient

Using the SEM module, the model in Figure was tested. This model had an acceptable fit to the data (R-CFI=.93, R-TLI=.92, R-RMSEA=.06, SRMR=.08). The eudaimonic motive positively predicts anonymous prosocial behavior (*β*=.49, *p*<.01) but does not significantly predict public prosocial behavior (*β*=.02, *p*=.864), so the first hypothesis is accepted. This finding aligns with previous research [32, 35], emphasizing that commitment to virtuous principles underpins the inclination of individuals with eudaimonic motives to engage in anonymous prosocial behavior. The positive association between eudaimonic motive and empathy, which emphasizes concern for others' needs, further supports this conclusion [54]. Individuals guided by eudaimonic motives conduct their activities in accordance with moral values and a commitment to the common good [55]. Eudaimonic individuals demonstrate a forward-looking perspective, considering the abstract consequences of actions, while also maintaining an awareness of society and the broader context. Simultaneously, they appreciate living in the present moment [56]. It is possible that individuals with eudaimonic motives may prefer anonymous prosocial behavior over public displays, as exposure to benevolent actions might be perceived as a violation of moral values [43]. Especially in Indonesia with strong religious nature among its society, recognition increased prosocial behavior only for those with high extrinsic religiosity, but not for those with high intrinsic religiosity [44]. The option to conceal or reveal their prosocial behavior becomes valuable for individuals who are less concerned with social approval, facilitating their engagement in prosocial acts [57].

Assisting others may come at a personal cost, particularly when individuals with eudaimonic motives are involved, as their prosocial actions might be subject to doubt or misunderstanding by others [42]. Notably, individuals guided by eudaimonic motives are known to exhibit superior emotion regulation, enabling them to handle negative emotions more effectively, a factor that has been linked to overall better wellbeing compared to individuals driven by hedonic motives [58]. The ability to regulate emotions becomes a crucial asset in navigating potential psychological and social costs associated with engaging in prosocial activities. For individuals pursuing eudaimonic motives for wellbeing, the fulfillment of their intention to live by moral values and contribute to others translates into enhanced wellbeing. Consequently, engaging in anonymous prosocial behavior is viewed as a means to assist others, uphold one's virtues, and simultaneously minimize the risk of others misjudging their actions.

Neither hedonic (*β*=-.11, *p*=.39) nor extrinsic motives (*β*=.12, *p*=.22) exhibit a significant negative association with anonymous prosocial behavior. Contrary to the second hypothesis, this hypothesis is rejected because hedonic motive has no effect on either public (*β*=-.11, *p*=.39) or anonymous prosocial behavior (*β*=.01, *p*=.93). This outcome might be attributed to the fact that individuals driven by hedonic motives, seeking pleasure and comfort, perceive helping others in both public and private settings as an additional burden and an extra expectation that detracts from personal enjoyment [34]. Individuals motivated by hedonic motives may view engaging in prosocial behavior, whether publicly or privately, as entailing both benefits and costs. Publicly, prosocial behavior may bring immediate positive mood rewards but can also incur resource costs and potential hassles. Opting for anonymous prosocial behavior may shield individuals from the risks associated with public assistance but also denies them potential rewards, even though they may be sacrificing personal comfort. For those driven by extrinsic motivation, assisting others in an anonymous setting carries a high risk of their actions going unrecognized and unrewarded.

As hypothesized, extrinsic motive emerges as a significant positive predictor of public prosocial behavior (*β*=.39, *p*<.01) while extrinsic motive does not significantly predict anonymous prosocial behavior (*β*=-.12, *p*=.22). The extrinsic motive, driven by a desire for personal benefits while helping others, makes public prosocial behaviors more likely to be acknowledged and praised. Consistent with prior research [40, 41], individuals with an extrinsic motive are inclined to engage in prosocial behavior within a public context, as this activity contributes to their impression management. Moreover, in collectivistic societies such as Indonesia, helping others is often perceived as a social norm. Individuals with an extrinsic motive for wellbeing may be motivated to seek social approval [59] by actively participating in public prosocial behaviors than in anonymous setting.

Engaging in activities centered around self-orientation, characterized by hedonic and extrinsic motives, is linked to psychological costs for one's health, social relationships, and overall wellbeing. This association arises from the potential anxiety and pressure individuals may feel in their efforts to uphold a particular impression for others [42]. Conversely, undertaking helpful behaviors with an other-oriented focus, as represented by eudaimonic motives, leads to an increase in positive mood, self-efficacy, a sense of meaning in life, and improved relatedness with others [60].

## Conclusion

This study revealed that the eudaimonic motive of wellbeing serves as a positive predictor of anonymous prosocial behavior, highlighting its influential role in shaping benevolent actions without seeking recognition. On the other hand, the extrinsic motive for wellbeing emerges as a positive predictor of engaging in public prosocial behavior. Interestingly, the hedonic motive has no discernible effect on either public or anonymous prosocial behavior. Notably, this research marks inaugural exploration of the distinct impacts of individuals' pursuit of wellbeing on their approaches to helping others. The underlying reason in seeking one’s wellbeing can promote or hinder to act prosocially in public or anonymous setting.

The current study was limited in its ability to determine why the hedonic motive for wellbeing did not significantly affect either public or anonymous behavior. Previous research has indicated that individuals driven by hedonistic motives may only extend help under specific conditions, such as when beneficiaries are their significant others, and when prosocial behavior has a high impact on minimal costs [34, 38]. Future investigations could delve deeper into potential moderators or mediators that shed light on how individuals with hedonistic motives for wellbeing may engage in public or anonymous prosocial behaviors. It is important to note that our cross-sectional design and reliance on convenience sampling among undergraduate students limit the ability to infer causality and generalize findings to the broader Indonesian population. Further experimental research is warranted to validate how motives for wellbeing influence different forms of prosocial behaviors. It is also recommended for further research to involve non-university-student participants. 

In terms of practical implications, for policy makers and institution supporting prosocial behavior, to engage university students with similar background with the present study, individuals who prioritize living virtuously and contributing to the common good, offering options to protect personal information and highlighting the impact of benefactors' actions on the lives of beneficiaries could be valuable. This can help build trust and encourage more individuals with eudaimonic motive to engage in prosocial activities. In anonymous settings, benefactors who receive information about the impact of their prosocial actions on beneficiaries tend to experience greater positive affect, lower negative affect, and a better sense of meaning in life than those who do not receive such information [61]. For those seeking rewards, a strategic approach could involve combining prosocial activities with recognition, monetary rewards, and appreciation, creating a shared narrative that enhances benefactors' personal branding. Overall, these recommendations can help create a more prosocial society where individuals are motivated to contribute to the common good.

## Data Availability

The datasets used and/or analyses during the current study are available from the corresponding author upon reasonable request.
